# Attitude of Nursing and Midwifery students towards clinical practice and its associated factors in Northwest Ethiopia: a cross-sectional study

**DOI:** 10.1186/s13104-019-4230-3

**Published:** 2019-04-03

**Authors:** Yosef Aragaw, Workinesh Sinishaw, Workinesh Daba, Maru Mekie

**Affiliations:** 1 Department of Midwifery, Debre Tabor Health Science College, Debre Tabor, Ethiopia; 2 Department of Nursing and Midwifery, College of Health Sciences, Addis Ababa University, Addis Ababa, Ethiopia; 3 Department of Midwifery, College of Health Sciences, Debre Tabor University, Debre Tabor, Ethiopia

**Keywords:** Nursing and Midwifery students, Attitude, Clinical practice

## Abstract

**Objective:**

The aim of this study was to assess the attitude of Nursing and Midwifery students towards clinical practice and its associated factors at University of Gondar, Northwest Ethiopia. 2018.

**Result:**

The prevalence of a favorable attitude towards clinical practice was found to be 42.9% in this study. The odds of having favorable attitude were found to be 2 times higher among students who prepared well for clinical practice [AOR = 2.07, 95%, CI (1.25, 3.44)] compared with counterparts. Similarly students who communicate well with clinical staffs [AOR = 1.89, 95%, CI (1.05, 3.41)], practiced in well-equipped hospital [AOR = 1.76, 95%, CI (1.01, 3.06)], and accompanying frequently by a clinical supervisor [AOR = 1.69, 95%, CI (1.02, 2.81)] were more likely to have favorable attitude compared with counterparts.

## Introduction

The meaningful correlation between theory and practical education is taking place in a clinical setting [1–5]. One of the bases for quality nursing service is nursing education which encompasses the three domains of learning; knowledge, attitude, and practice [6, 7].

Attitude plays a major role in leading human toward achieving the desired goals, awareness of its consequences, and effective processing of complex information [5, 6, 8]. Students’ attitude towards clinical practice could be affected by a clinical environment, student staff interaction, clinical preceptors, and the availability of necessary equipment in a hospital setting [9, 10].

Lack of interest in what people do could decrease the quality of work [5, 6, 11]. More than half of all healthcare providers are Midwives and Nurse in the healthcare setting [5, 6]. Hence, if the attitude of Nursing and Midwifery professionals is not favorable, the quality of health care could be compromised in a significant manner. In Ethiopia, students join Nursing and Midwifery degree programs directly after completing secondary education which demands intensive clinical training to acquire Nursing and Midwifery competencies [12].

There is a significant incoherence between theory and clinical practice [9] and one of the cause of this discrepancy could be attitude. A positive attitude towards clinical practice enhances effective clinical learning. Whereas, a negative attitude hampers the acquisition of essential clinical skills. Thus, identifying the gap in clinical practice is noteworthy for improving the quality of Nursing and Midwifery educations [13].

## Main text

### Methods

#### Study design and population

An institution based cross-sectional study was employed from March 19 to 26, 2018 on 345 Nursing and Midwifery students at University of Gondar. The University of Gondar (UoG), until 2003 known as Gondar College of Medical Sciences. It is the oldest medical school in Ethiopia which was established as the Public Health College in 1954. It is also the pioneer institution to initiate degree Midwifery training program in Ethiopia [12, 14]. The study population was randomly selected regular Nursing and Midwifery students who fulfill the inclusion criteria.

#### Inclusion and exclusion criteria

Randomly selected second-year and above regular Nursing and Midwifery students and those who were willing to participate in the study were included. Whereas, all first-year regular Nursing and Midwifery students, and those refused to participate in the study were excluded.

#### Sample size determination

**Fig. 1:**
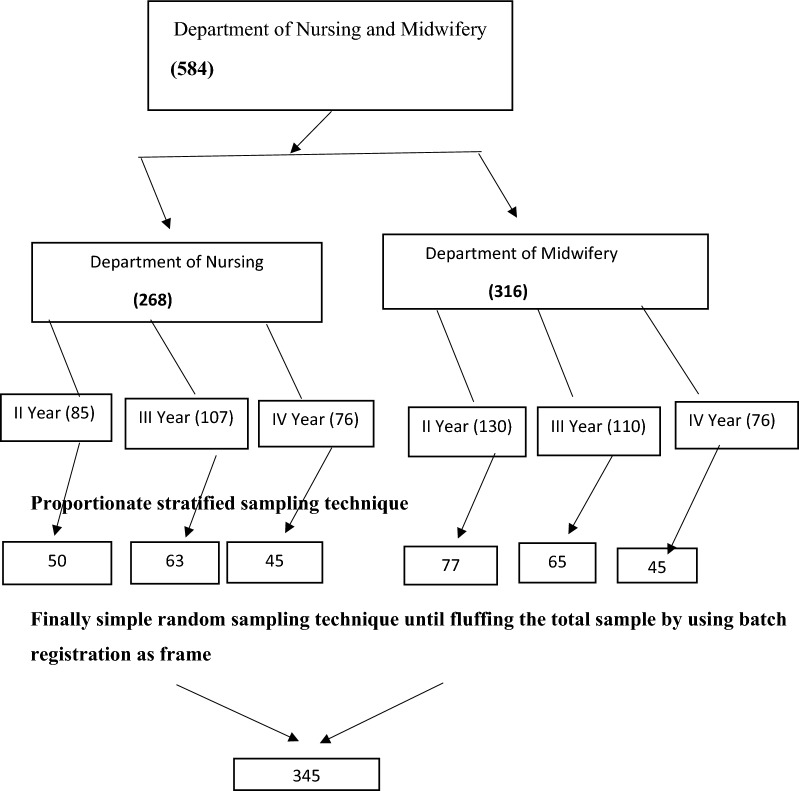
Sampling procedure for selection of the study participants

A single population proportion formula was used to calculate sample size at Zα/2 = 1.96, 95% confidence interval, d = 0.05 degree of margin of error, and proportion (P = 0.5 since there was no similar study as far as the authors’ knowledge). Thus, the final sample size after adding 10% non-response rate was 345 by using a correction formula (Fig. 1).

#### Measurement of variables and definitions

The attitude towards clinical practice was measured based on the mean of the sum of fifteen attitude questions which was 9.41 points with minimum and maximum scores of 2 and 15 points, respectively. The attitude score was dichotomized as favorable and unfavorable. Attitude scores ≤ 9.41 was taken as “unfavorable attitude” while > 9.41 was taken as “favorable attitude.”

Well-equipped hospital: Is a hospital which provides all services to be provided at a hospital level using the current available laboratory setting. Adequacy of rooms were also considered in this study.

Accompanied frequently: In this study accompanied frequently by a clinical supervisor is considered if the clinical supervisor is available with students at least 3 days a week.

Communicate well: Students who interact with staffs in every procedure were considered as communicate well with staffs in this study.

#### Data collection process and quality assurance

The calculated sample was allocated to the selected Departments based on the number of students per years of study. Then study participants were selected by a simple random sampling technique from each year of study using students’ registration. Data were collected by a self-administered questionnaire prepared in English after orienting the selected participants. The questionnaire was adapted from previously published literature [1, 3, 7, 15, 16]. Content validity of each questionnaire was evaluated based on the review of nine experts in the field of Nursing and Midwifery. Experts’ ratings were used to calculate the content validity index (CVI) values of each item. Each item with validity index > 0.78 was included in the final instrument.

#### Data analysis and processing

Data was entered by EpiData version 3.1 and analyzed by SPSS version 20. Bivariable and multivariable logistic regressions were used to identify the significance, strength, and direction of the association at 95% confidence interval. An adjusted odds ratio were used to identify the independent predictors of attitude. All factors with a P value of < 0.2 in the bivariable analysis were entered in the multivariable model to identify the independent predictors of attitude towards clinical practice. *P* value of < 0.05 was used to decide the significance of association at 95% confidence interval.

#### Ethical consideration

Ethical approval and letter of permission were obtained from the College of Health Science, AAU. The letter of permission was submitted to the Nursing and Midwifery Departments, College of Medicine and Health Sciences, UoG to get permission. Following the permission, participants were briefed about the study purpose and the procedure of the data collection process. The privacy of the study participants’ and confidentiality of information was maintained.

### Result

#### Socio-demographic characteristics of study participants

A total of 345 students from the two Departments were participated in the study. Five questionnaire were excluded from the analysis due to gross incompleteness of responses, giving a response rate of 98.6%. Nearly three forth of the study participants were found in age group of 20–24 years (Table 1).

**Table 1:** Socio-demographic characteristics of Nursing and Midwifery students at UoG, Northwest Ethiopia, 2018

Characteristics	Frequency	Percent (%)
Sex (N = 340)		
Male	170	50.0
Female	170	50.0
Age in years (N = 340)		
15–19	73	21.5
20–24	246	72.3
25–29	19	5.6
≥ 30	2	0.6
Assigned in clinical practice in your stay (N = 340)		
One	81	23.8
Two	93	27.4
Three	63	18.5
Four and above	103	30.3
Year of study (N = 340)		
2nd year	125	36.8
3rd year	125	36.8
4th year	90	26.4
Field of study (N = 340)		
Nursing	155	45.6
Midwifery	185	54.4
Do you use substance? (N = 340)		
Yes	20	5.9
No	320	94.1
Type of substance (N = 20)		
Chat	6	1.8
Alcohol	12	3.5
Cigarette	2	0.6
Frequency/week N = 20		
Daily	2	1.8
2–4 day/week	11	3.5
Once a week	7	0.6

#### Factors affecting students’ attitude towards clinical practice

Table 2 below revealed the multivariable analysis of factors affecting the attitude of students towards clinical practice among students in UoG. The odds of having favorable attitude were found to be 2 times higher among students who prepared well for clinical practice [AOR = 2.07, 95%, CI (1.25, 3.44)] compared with counterparts.

**Table 2:** Multivariate logistic regression analysis of predictors of attitude towards clinical practice, in UoG, 2018

Variable	Attitude to clinical practice		COR (95% CI)	AOR (95% CI)
	Favorable	Unfavorable		
Time of assigned in clinical practice				
One	29 (19.9%)	52 (26.8%)	1	1
Two	35 (24%)	58 (29.9%)	1.08 (0.58, 2.01)	1.91 (0.84, 4.33)
Three	28 (19.1%)	35 (18.0%)	1.43 (0.73, 2.81)	2.93 (0.91, 9.48)
Four and above	54 (37%)	49 (25.3%)	1.98 (1.09, 3.59)*	2.08 (0.54, 7.98)
Year of study				
2nd	50 (34.2%)	75 (38.7%)	1	1
3rd	41 (28.1%)	84 (43.3%)	0.73 (0.44, 1.23)	0.45 (0.18, 1.12)
4th	55 (37.7%)	35 (18.0%)	2.36 (1.35, 4.11)*	1.21 (0.33, 4.45)
Prepared for clinical practice				
Yes	69 (47.3%)	68 (35.1%)	1.66 (1.07, 2.58)*	2.07 (1.25, 3.44)**
No	77 (52.7%)	126 (64.9%)	1	1
Communicate well with staff				
Yes	119 (81.5%)	139 (71.6%)	1.74 (1.04, 2.94)*	1.89 (1.05, 3.41)**
No	27 (18.5%)	55 (28.4%)	1	1
Ready to interact with staffs				
Yes	132 (90.4%)	151 (77.8%)	2.69 (1.41, 5.13)*	2.02 (0.97, 4.21)
No	14 (9.6%)	43 (22.2)	1	1
Staff show respect to students				
Yes	103 (70.5%)	118 (60.8%)	1.54 (0.98, 2.44)	0.82 (0.47, 1.45)
No	43 (29.5%)	76 (39.2%)	1	1
Favorable hospital				
Yes	100 (68.5%)	96 (49.5%)	2.22 (1.42,3.48)*	1.76 (1.01, 3.06)**
No	46 (31.5%)	98 (50.5%)	1	1
Sufficient material in clinical area				
Yes	93 (63.7%)	95 (49.0%)	1.83 (1.18, 2.84)*	1.17 (0.69, 1.99)
No	53 (36.3%)	99 (51.0%)	1	1
Attended by supervisor frequently				
Yes	82 (56.2%)	76 (39.2%)	1.99 (1.29,3.08)*	1.69 (1.02,2.81)**
No	64 (43.8%)	118 (60.8%)	1	1
The instructor facilitates staff relationship				
Yes	125 (85.6%)	134 (69.1%)	2.67 (1.53, 4.64)*	1.63 (0.85, 3.11)
No	21 (14.4%)	60 (30.9%)	1	1
The instructor provides me constructive feedback				
Yes	101 (69.2%)	110 (56.7%)	1.71 (1.09, 2.69)*	1.25 (0.73, 2.12)
No	45 (30.8%)	84 (43.3%)	1	1
My pleasure is to meet patient needs				
Yes	142 (97.3%)	193 (99.5%)	0.18 (0.02, 1.66)	0.22 (0.02, 2.64)
No	4 (2.7%)	1 (0.5%)	1	1
Maintain patient dignity				
Yes	129 (88.4%)	183 (94.3%)	0.46 (0.21, 1.01)	0.51 (0.21, 1.28)
No	17 (11.6%)	11 (5.7%)	1	1

NB: * Significate in the binary analysis; ** Significant in the multivariable analysis

*CI* confidence interval, *COR* crude odds ratio, *AOR* adjusted odds ratio

### Discussion

The prevalence of favorable attitude towards clinical practice was 42.9% in this study which indicates more than half, (57.1%) of the study participants had an unfavorable attitude. Unfavorable attitude can lead to poor intent to attend theory and practical sessions [6, 17]. Consistent with a previous study [1], seniority was found to be associated with having a favorable attitude towards clinical practice in our study, 55 (37.7%) and 50 (34.2%) among 4th and 2nd-year students, respectively. Similar with previous studies [18–20], the majority of students (97.9%) agreed that clinical practice is the major area of Nursing and Midwifery profession in this study. The multivariable analysis revealed that the odds of having a favorable attitude towards clinical practice were found to be 2 times higher among students who prepared well compared with counterparts [AOR = 2.07, 95%, CI (1.247, 3.439)]. Appropriate preparation before clinical attachment increases students’ desire to learn in a clinical setting. The finding is consistent with a study done in Norway [21].

With regards to interaction with staffs, the odds of having a positive attitude towards clinical practice were found to be 2 times higher among students who had good communication with clinical staffs [AOR = 1.89, 95%, CI (1.05, 3.41)] compared counterparts. The finding of our study is consistent with previous studies conducted in Jamaica [22], Spain [2], and Iran [3]. This might be due to the fact that clinical staffs are the key stakeholders to shape students with necessary skills, creating conducive clinical-environment, and socializing students with their profession [23]. Similarly, students who practiced in a well-equipped hospital were more likely to have a favorable attitude towards clinical practice compared with counterparts [AOR = 1.76, 95%, CI (1.01, 3.06)]. This might be due to the fact that students could have a better picture of the clinical setup which derives the learning objectives. The finding is supported by the studies done in Australia [8], and Afghanistan [15].

The multivariable analysis indicated that the odds of having favorable attitude were found to be 1.7 times higher among students who were frequently accompanied by clinical-mentors compared with counterparts [AOR = 1.69, 95%, CI (1.018, 2.805)]. This might be due to the fact that clinical mentor can smoothen the relationship between clinical staff and students by bridging gaps. The finding is consistent with the studies done in Norway [21], Iran [24], and Rwanda [23].

### Conclusion

Students who had good communications with clinical staffs, prepared well for clinical practice, practiced in well-equipped hospital, and accompanied frequently by clinical-supervisors in a clinical setting had a favorable attitude towards clinical practice.

## Limitation

The study design is cross-sectional which might be difficult to make cause and effect relationship. Using only a quantitative method might not explicate the real attitude of students related to clinical practice. We have tried to collect data based on the immediate clinical experience of students’ which can be taken as a strength.

## Abbreviations

AAU: Addis Ababa University
CI: confidence interval
UoG: University of Gondar
